# Elastofibroma developing at the subscapular port site after thoracoscopic surgery: first case report

**DOI:** 10.1186/s40792-017-0398-8

**Published:** 2017-12-06

**Authors:** Chihiro Yoshida, Noriyuki Misaki

**Affiliations:** Department of General Thoracic Surgery, Takamatsu Municipal Hospital, 2-36-1 Miyawakichou, Takamatsu, Kagawa 760-8538 Japan

**Keywords:** Elastofibroma, Thoracoscopic surgery, Subscapular tumor, Port-site cancer recurrence

## Abstract

In recent years, the number of cancer patients who undergo endoscopic surgery has been increasing, and port-site recurrence is becoming a more common complication. A 66-year-old woman underwent thoracoscopic left lower lobectomy with lymph node dissection for pT1aN0M0 adenocarcinoma of the lung. Six years after surgery, CT revealed a subscapular tumor measuring 3 cm at the site of the surgical port wound. Although port-site cancer recurrence was suspected, needle biopsy revealed that the tumor was an elastofibroma. During 6 months of follow-up, MRI revealed no further change, and it was concluded that development of the tumor at the subscapular port site had been merely coincidental.

## Background

Elastofibromas are benign, connective tissue tumors that grow slowly. They frequently arise between the inferior corner of the scapula and the posterior chest wall. These tumors are infrequent, representing 1–2% of all primary tumors of the chest wall [1]. Here we report a primary elastofibroma that developed at the subscapular port site after thoracoscopic surgery for lung cancer, necessitating differentiation from port-site recurrence of the cancer.

## Case presentation

**Fig.1:**
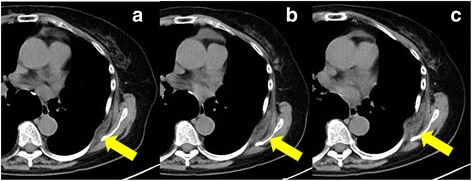
**a** CT scan at 4 years after surgery shows a subscapular mass with a density similar to that of the skeletal muscle with moderate enhancement. **b**, **c** CT scans at 5 and 6 years after surgery shows gradual growth of the subscapular mass over time

**Fig.2:**
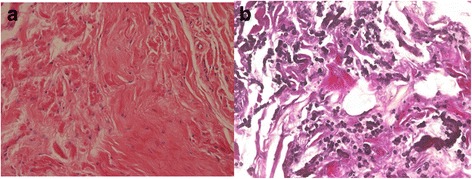
**a** Hypocellular fibrocollagenous tissue is visible (H&E ×20). **b** Thick or coarse elastic fibers arranged in beaded strings or globules are demonstrated using elastica van Gieson staining (×20)

In 2011, a 66-year-old housewife patient underwent thoracoscopic left lower lobectomy and lymph node dissection for lung cancer. In general, a 10-mm, 30-degree thoracoscope was placed through the intercostal space in the midaxillary line and a 2-cm incision was then made in the seventh intercostal space just beside the scapular angle. Subsequently, an access thoracotomy was always located in the fourth intercostal space in the anterior axillary line. The histopathological diagnosis was adenocarcinoma stage IA3 (pT1cN0M0) (tumor invasive size, 2.1 × 1.3 cm). At 6 years of follow-up as an outpatient, CT revealed a fusiform subscapular soft tissue heterogeneous solid mass with muscle-like density measuring 30 × 35 mm at the site of the subscapular surgical port wound (Fig. 1). Retrospective examination of CT films revealed slow growth of the tumor. Port-site recurrence of the lung cancer was suspected, and US-guided biopsy of the tumor was performed. Pathologically, the tumor was composed of hypocellular fibrous collagenous strands admixed with large numbers of coarse, densely eosinophilic elastic fibers, and there was no malignancy (Fig. 2). Elastica van Gieson staining demonstrated extending and globoid elastic fibers. These findings were consistent with elastofibroma.

## Discussion and conclusions

Elastofibroma is a rare, benign, slow-growing soft tissue tumor that was first defined by Jarvi and Saxen in 1961 [2]. Elastofibroma occurs more commonly in older women, with a reported female/male ratio of 5:1 and a mean age at diagnosis of about 60 years. Ninety-nine percent of elastofibromas are located in the subscapular region and more commonly on the right; however, about 25% of cases are bilateral [3–5]. The pathogenesis of elastofibroma is unclear, but three etiological theories have been proposed [6, 7]. As the lesion was first observed among workers performing heavy manual labor, the causative mechanism proposed was repetitive friction of the scapula against the thorax, triggering overproduction of collagenous connective tissue with degeneration of collagen fibers, associated with production of excessive amounts of elastic matrix alternating with deposition of hyperplastic fat. Another theory for the pathogenetic sequence was reactive fibromatosis and degeneration secondary to vascular insufficiency, with elastic degeneration. Finally, a familial predisposition due to an underlying enzymatic defect was proposed by Fukuda et al. as a possible etiologic factor [6, 8, 9]. In a study based in Okinawa Prefecture, Japan, Nagamine et al. noted that 32% of their cases occurred within a single family [9].

Elastofibromas have characteristic imaging features on MRI and CT that allow definitive diagnosis. In most cases, CT shows poorer differentiation of tumor edges from the surrounding muscle planes in comparison with ultrasonography and MRI: the tumor appears as a semilunar mass of soft tissue with a density similar to that of the adjacent musculature, containing linear areas of low density secondary to fat. MRI is a non-invasive imaging modality with the greatest sensitivity and specificity for detection of elastofibromas because it demonstrates a circumscribed mass with an alternating pattern of fibrous and fatty tissues. On MRI, a typical feature of elastofibroma is that the interposed areas of decreased signal intensity also appear as low signal intensity on T2-weighted sequences [10–12]. Histologically, the diagnosis is based on the presence of a mixture of paucicellular collagenous tissue and a large number of elastic fibers, associated with a small amount of mucoid stroma and entrapped mature fat cells. The elastic fibers are often large, coarse, deeply eosinophilic and fragmented into small, linearly arranged globules or serrated disks simulating beads on a string [8, 13]. Generally, conservative treatment is recommended for elderly and asymptomatic patients; in fact, to date, malignant transformation has never been reported [11]. On the other hand, the indications for surgery depend on the severity of the symptoms and patient preference. Marginal excision is sufficient, and surgery may achieve dramatic relief of troublesome symptoms.

We report a patient in whom CT demonstrated a subscapular pleural tumor at the site of the surgical port wound 6 years after thoracoscopic surgery for lung cancer. Retrospectively, CT showed that the tumor had grown slowly for two years, and therefore, we suspected port-site recurrence or a benign soft tissue tumor such as lipoma, hemangioma, or desmoid tumor. As we were particularly concerned about port-site recurrence, diagnostic needle biopsy was performed. Although MRI, which is considered the main imaging technique for diagnosis, clearly demonstrated a heterogeneous ill-defined lesion with an alternating pattern of fibrous and fatty tissue [14], in the present case, diagnosis was based on needle biopsy, and therefore, MRI was not performed. The most common site of elastofibroma is the subscapular region, which is generally the site of port placement for thoracoscopic excision of lung cancer. There have been no previous reports of elastofibroma developing at a subscapular port site after thoracoscopic surgery. Although the pathogenesis remains unclear, certain mechanisms may have been responsible for the development of elastofibroma in the present context. Since elastofibroma is suggested to be a proliferative response of connective tissue to mechanical stress such as repetitive trauma or manual labor [15], thoracoscopic surgery may have been a factor in the present case. It is possible that the popularization of thoracoscopic surgery may lead to an increase in the incidence of elastofibroma at the subscapular port site. Therefore, the present case suggests that in patients who have undergone thoracoscopic surgery for lung cancer, any tumor developing in the subscapular region needs to be differentiated from elastofibroma.

We hope that the present case will add to the growing body of clinical experience, stimulate further investigation, and ultimately lead to better patient outcomes.

### The study design

The significance of differentiation elastofibroma developing at the subscapular port site after thoracoscopic surgery from port-site recurrence is studied.
